# Exacerbation of Secondary Cold Agglutinin Syndrome in the Setting of SARS-CoV-2

**DOI:** 10.7759/cureus.19387

**Published:** 2021-11-09

**Authors:** Yash V Bhagat, Siham Hussien, Helen Queenan, Miriam B Michael

**Affiliations:** 1 Internal Medicine, University of Maryland Midtown Campus, Baltimore, USA; 2 College of Medicine, American University of Antigua, St. John's, ATG; 3 Internal Medicine, Howard University College of Medicine, Washington, D.C., USA

**Keywords:** transfusion in covid 19 pandemic, severe acute respiratory syndrome coronavirus-2 (sars-cov-2), covid hypercoagulability, covid-19 associated coagulopathy, covid-19 pneumonia, covid coagulopathy, autoimmune hemolytic anemia (aiha), cold agglutinin syndrome, sars-cov-2, covid-19

## Abstract

In this report, we present a case of exacerbation of cold agglutinin syndrome (CAS) potentially due to severe acute respiratory syndrome coronavirus 2 (SARS-CoV-2) pneumonia. An 83-year-old female with a history of cold agglutinin hemolytic anemia presented with shortness of breath, productive cough, worsening orthopnea, darkening fingers and urine, and jaundice. Laboratory investigations found elevated white blood cells (WBC) and total bilirubin, severely low hemoglobin, and positive direct Coombs test. Moreover, SARS-CoV-2 RNA was also found to be positive in a sample from the nasal swab by reverse transcription-polymerase chain reaction (RT-PCR), indicating exacerbation of CAS secondary to viral coronavirus 2019 (COVID-19) infection. A treatment regime for SARS-CoV-2 consisting of five days of remdesivir and seven days of dexamethasone 6 mg IV was initiated, resulting in significant improvement in the patient's condition.

## Introduction

Autoimmune hemolytic anemias (AIHAs) constitute a group of diseases characterized by the production of autoantibodies directed to antigens on red blood cells (RBCs). The three arms of AIHAs are warm, cold, and mixed AIHAs. Cold AIHAs are further classified into primary cold agglutinin disease (CAD), paroxysmal cold hemoglobinuria, and secondary CAD or cold agglutinin syndrome (CAS). The phenomenon of agglutination of blood at cold temperatures was first noted in the 19th century and the term “cold agglutinin disease” was coined in 1966 by Schubothe [1]. Cold agglutinins react optimally at temperatures of 0-4 ℃ (32-39 °F) but may also be reactive at a wider thermal amplitude that may occur physiologically in the extremities of patients, likely presenting with acrocyanosis of the nose, ears, fingers, and toes. Pentameric immunoglobulin M (IgM) antibodies, the cause of about 90% of CAS cases as compared to IgG and IgA, bind to antigens on the surface of RBCs, thereby activating the complement pathway leading to extravascular or intravascular hemolysis depending on whether complement activation progresses past the C3 step to initiate the membrane attack complex [1,2]. The diagnosis of CAS is made by positive direct antiglobulin testing, positive anti-C3d testing, and a cold agglutinin titer of at least 1:64 [1]. CAS may occur secondary to underlying infections. Some known infections associated with secondary CAS are those caused by the Epstein-Barr virus and *Mycoplasma pneumoniae* [3,4]. Severe acute respiratory syndrome coronavirus 2 (SARS-CoV-2), an enveloped positive-sense single-stranded RNA virus of the Coronaviridae family that is transmitted through respiratory droplets, is thought to infect the respiratory tract, gastrointestinal tract, cardiac muscle, and endothelial cells by binding to the angiotensin-converting enzyme 2 receptors [5]. SARS-CoV-2 infections present with symptoms ranging from fever, dry cough, sore throat, and runny nose to severe pneumonia, hypoxia, and acute respiratory distress syndrome (ARDS). Additionally, recent studies of the SAR-CoV-2 virus have revealed a putative association between the virus and CAS, which we explore further in this report [6].

## Case presentation

**Figure 1:**
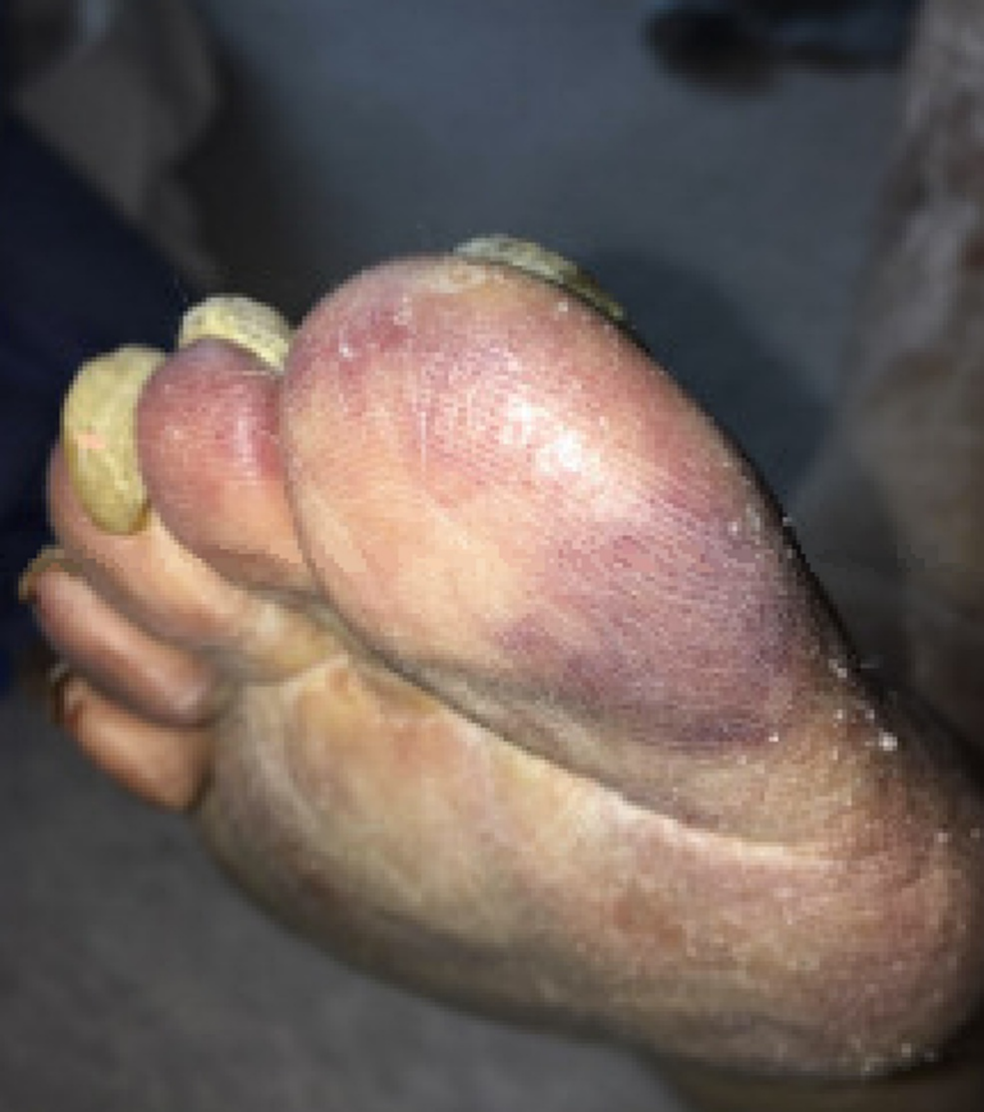
Acrocyanosis of the patient's toe due to cold agglutinin hemolytic anemia

An 83-year-old female with a history of cold agglutinin hemolytic anemia requiring transfusions, chronic anemia with hemoglobin levels between 7-8 g/dl, hypothyroidism, hypertension, deep vein thrombosis in bilateral lower extremities, and chronic lymphedema, presented with shortness of breath, cough, weakness, lightheadedness, acrocyanosis or darkening of the fingers and toes (Figure 1), jaundice, and darkening of her urine. She was found to have a productive cough with yellow sputum and worsening shortness of breath for the last four days, along with progressively worsening orthopnea that required sleeping upright. However, the patient denied chest pain, worsening leg swelling, paroxysmal nocturnal dyspnea, and wheezing.

On presentation, the patient was afebrile, normotensive, with a normal heart rate, but had severe hypoxia with a saturation of 88% on room air and 95% with 2 liters of oxygen administered via nasal cannula. Throughout hospitalization, her temperature ranged between 96.7 and 98.6 °F (36-37 ℃). Physical examination was remarkable for mucosal pallor, acrocyanosis, icterus of the facial skin, diffuse bilateral rhonchi on lung auscultation, and pitting edema in bilateral lower extremities below the knee. Chest X-ray showed an enlarged cardiac silhouette, perihilar vascular fullness, and bilateral interstitial prominence likely indicative of pulmonary vascular congestion. A cardiac echocardiogram found the ejection fraction to be 60-65% with mild left ventricular wall thickness and grade I diastolic dysfunction. However, B-type natriuretic peptide (BNP) levels were only mildly elevated at 571 pg/mL. Moreover, nasal swab testing for SARS-CoV-2 was positive but was negative for influenza A, influenza B, and respiratory syncytial virus.

Complete blood count revealed a high white blood count of 26.8 thousand/uL (normal range: 4.0-10.8 thousand/uL), low hemoglobin of 5.8 g/dl (12.0-16.0 g/dL), low haptoglobin of 1 mg/dL (41-165 mg/dL), and high erythrocyte sedimentation rate of 110 mm/hr (0-29 mm/hr). Serum chemistry testing found hyperkalemia of 6.7 mEq/L (3.5-5.1 mEq/L), elevated blood urea nitrogen of 34 mg/dl (8-25 mg/dl) with normal creatinine of 0.6 mg/dl (0.6-1.3 mg/dl), and severely elevated total bilirubin of 10.8 mg/dL (0.1-1.4 mg/dL). Blood culture collected from two different sites revealed no growth. Direct Coombs test was positive, and direct Coombs C3D test was also found to be positive during this admission. Testing from prior admissions had determined a cold agglutinin titer of 1:512. Other relevant testing included negative findings on hepatitis B virus IgM core antibodies, hepatitis B virus surface antigen, and hepatitis C virus antibody.

To treat the symptomatic anemia, the patient was transfused with two units of crossmatch least incompatible warmed blood on day one and one unit of the same on day two of admission. The SARS-CoV-2 pneumonia was treated with oral remdesivir for five days and intravenous dexamethasone 6 mg for seven days. The patient was also given intravenous 40 mg furosemide for pulmonary congestion, and empiric ceftriaxone and azithromycin for pneumonia for three days. Post-transfusions, the patient’s day-three hemoglobin was 10.2 g/dL, which dropped in the next few days but stabilized at her baseline of 7-8 g/dL by discharge on day 10. By day three, the patient’s hyperkalemia had improved without intervention; the furosemide was discontinued since the patient was euvolemic on examination, and the azithromycin and ceftriaxone were stopped due to low concern for concomitant bacterial pneumonia. The patient remained in stable condition in the designated isolation room through the rest of her hospital stay, and after 10 days since her initial positive coronavirus 2019 (COVID-19) test, the isolation protocol was lifted. She reported feeling like she was “regaining her strength”. Yet, her oxygen levels persisted to desaturate with exertion and an ambulatory pulse oximetry examination revealed that she required 3 L of oxygen on discharge. The patient was recommended to be discharged to a sub-acute rehabilitation center.

Two days post-discharge, the patient had another drop in her hemoglobin to 6.3 g/dL and required admission to a cancer institute where she was hospitalized for eight days, requiring five units of packed red blood cells and 60 mg of methylprednisolone IV daily. She had no signs and symptoms of bleeding during her admission. Her hemoglobin level improved to 9.2 g/dl and remained stable prior to discharge. On evaluation by an oncologist, weekly treatment with rituximab was recommended post-discharge for four weeks. On discharge, she was also prescribed prednisone 40 mg for seven days followed by a taper to 20 mg for another seven days and a follow-up appointment with oncology.

## Discussion

We reported a case of CAS exacerbation with the production of cold agglutinins secondary to SARS-CoV-2 or COVID-19 infection. Our patient presented with severe shortness of breath and hypoxia. Hence, some possible differential diagnoses that we chose to rule out included pneumonia, heart failure, myocardial infarction, and COVID-19 infection. After careful investigation and consideration for other causes of severe shortness of breath and hypoxia, the diagnosis of cold agglutinin hemolytic anemia exacerbation secondary to COVID-19 infection was confirmed with a positive direct Coombs test, positive direct Coombs C3D test, positive nasal swab for SARS-CoV-2, and lab results showing evidence of hemolytic anemia.

CAD is a B-cell monoclonal lymphoproliferative disorder, whereas CAS is a polyclonal B-cell disorder. The cold agglutinins produced by the monoclonal B cells in CAD have the propensity to be more pathogenic than the polyclonal cold agglutinins in CAS. The majority of the antibodies produced are IgM, which tends to bind red blood cell surface, activate complement, and facilitate hemolysis [7]. We hypothesize that infections with the SARS-CoV-2 virus, which further activate complement and upregulate anaphylatoxins C3a and C5a, may amplify the hemolysis caused by cold agglutinins [8]. Therefore, COVID-19 infections, similar to infections with Epstein-Barr virus and *Mycoplasma pneumoniae*, may be an independent risk factor predisposing individuals to the exacerbation of CAS.

There have been cases of cold agglutinins produced secondary to COVID-19 infection in the literature, where further serological studies have shown the presence of anti-I antibodies, which are found post-infection with *Mycoplasma* and H1N1. The presence of anti-I antibodies complicates the course of recovery [9]. Studies focusing on the treatment of patients with CAS exacerbation secondary to COVID-19 have reported successful resolution of CAS with rituximab and disease-modifying antirheumatic drugs (DMARDS). Additionally, using corticosteroids aids in the treatment of SARS-CoV-2 pneumonia [10]. The use of therapeutic plasma exchange (TPE) to remove the pathologic IgM autoantibodies and assist in the recovery from the COVID-19 infection has also been shown to be efficacious [11]. However, currently, there is no evidence-based treatment for CAS secondary to COVID-19 infection. The treatment of our patient was successful with a combination of transfusions, corticosteroids, and monoclonal antibodies. The patient’s severe anemia may also have benefited from TPE. Additionally, further antibody testing for the anti-I antibodies might have been beneficial for the detection of concurrent infection with *Mycoplasma pneumoniae* [12]. Further workup, including testing for D-dimer, prothrombin time, partial thromboplastin time, fibrinogen, and serum protein electrophoresis, can help inform treatment. Further research to establish evidence-based screening to inform treatment for CAS secondary to COVID-19 infection is necessary to ensure adequate resolution and decrease complications such as thromboembolic events and exacerbation of autoimmune disorders [13]. We believe that future treatments for CAS secondary to COVID-19 might involve monoclonal therapies targeting complement, like eculizumab, or C1 esterase inhibitors, like Sutimlimab [14].

## Conclusions

Our patient presented with exacerbation of cold agglutinin hemolytic anemia and concurrent infection with COVID-19. The lack of triggers for the exacerbation of her hemolytic anemia, along with the severe hypoxia due to her SARS-CoV-2 pneumonia, was putatively indicative of secondary CAS. Association between COVID-19 and CAS is a recent and novel finding. The lack of established evidence-based treatments for the disorder warrants further research in this area of medicine.
